# West Nile Virus and Other Nationally Notifiable Arboviral Diseases — United States, 2016

**DOI:** 10.15585/mmwr.mm6701a3

**Published:** 2018-01-12

**Authors:** Alexis Burakoff, Jennifer Lehman, Marc Fischer, J. Erin Staples, Nicole P. Lindsey

**Affiliations:** ^1^Epidemic Intelligence Service, CDC; ^2^Arboviral Diseases Branch, Division of Vector-Borne Diseases, National Center for Emerging and Zoonotic Infectious Diseases, CDC.

Arthropod-borne viruses (arboviruses) are transmitted to humans primarily through the bites of infected mosquitoes and ticks. West Nile virus (WNV) is the leading cause of domestically acquired arboviral disease in the continental United States (*1*,*2*). Other arboviruses, including La Crosse, Powassan, Jamestown Canyon, St. Louis encephalitis, and eastern equine encephalitis viruses, cause sporadic cases of disease and occasional outbreaks. This report summarizes surveillance data reported to CDC for 2016 for nationally notifiable arboviruses. It excludes dengue, chikungunya, and Zika viruses, as these are primarily nondomestic viruses typically acquired through travel. Forty-seven states and the District of Columbia (DC) reported 2,240 cases of domestic arboviral disease, including 2,150 (96%) WNV disease cases. Of the WNV disease cases, 1,310 (61%) were classified as neuroinvasive disease (e.g., meningitis, encephalitis, acute flaccid paralysis), for a national incidence of 0.41 cases per 100,000 population. After WNV, the most frequently reported arboviruses were La Crosse (35 cases), Powassan (22), and Jamestown Canyon (15) viruses. Because arboviral diseases continue to cause serious illness, maintaining surveillance is important to direct prevention activities.

Arboviruses are maintained in a transmission cycle between arthropods and vertebrate hosts, including humans and other animals. Humans primarily become infected when bitten by an infected tick or mosquito. Person-to-person transmission through blood transfusion and organ transplantation has been reported but is uncommon (*3*). Most human infections are asymptomatic; symptomatic infections commonly manifest as a systemic febrile illness and, less commonly as neuroinvasive disease.

Most endemic arboviral diseases are nationally notifiable and are reported to CDC through ArboNET, a national arboviral surveillance system managed by CDC and state health departments (*3*,*4*). Using standard definitions, human cases with laboratory evidence of recent arboviral infection are classified as having either neuroinvasive or non-neuroinvasive disease (*3*). Cases reported as encephalitis, meningitis, acute flaccid paralysis, or other neurologic manifestations were categorized as neuroinvasive disease. Reports without indication of a central neurologic process were categorized as non-neuroinvasive disease. Acute flaccid paralysis can occur with or without encephalitis or meningitis. In this report, any case reported as acute flaccid paralysis (with or without another clinical syndrome) was classified as acute flaccid paralysis and not included in the other categories. Because ArboNET is a passive surveillance system, detection and reporting of neuroinvasive disease are thought to be more consistent and more complete than non-neuroinvasive disease, which is likely considerably underreported. For this reason, incidence rates were calculated using neuroinvasive disease cases and the U.S. Census 2016 mid-year population estimates.

In 2016, 2,240 cases of domestic arboviral diseases were reported to CDC. Cases were caused by WNV (2,150 cases, 96%), La Crosse virus (35), Powassan virus (22), Jamestown Canyon virus (15), St. Louis encephalitis virus (eight), eastern equine encephalitis virus (seven), and unspecified California serogroup virus (three). Of the 3,142 U.S. counties, 656 (21%) reported one or more cases of arboviral disease. No cases of domestic arboviral disease were reported from Alaska, Hawaii, or Delaware.

Overall, 2,150 WNV disease cases were reported from 604 counties in 45 states and the District of Columbia. Of these, 1,310 (61%) cases were neuroinvasive and 1,781 (83%) patients had illness onset during July–September (Table 1). The median age of patients was 57 years (interquartile range [IQR] = 44–68 years); 1,326 (62%) were male. A total of 1,465 (68%) patients were hospitalized and 106 (5%) died. The median age of patients who were hospitalized was 61 years (IQR = 48–72 years) and 947 (65%) were male. The median age of patients who died was 75 years (IQR = 62–82 years) and 63 (59%) were male.

**TABLE 1:** Number and percentage of reported cases of West Nile virus and other arboviral diseases, by virus type and selected patient characteristics — United States, 2016*

Characteristic	Virus type, no. (%)					
	West Nile	La Crosse	Powassan	Jamestown Canyon	Saint Louis encephalitis	Eastern equine encephalitis
	(N = 2,150)	(N = 35)	(N = 22)	(N = 15)	(N = 8)	(N = 7)
**Age group (yrs)**						
<18	61 (3)	28 (80)	1 (5)	0 (0)	0 (0)	1 (14)
18–59	1,152 (54)	5 (14)	2 (9)	7 (47)	4 (50)	2 (29)
≥60	937 (44)	2 (6)	19 (86)	8 (53)	4 (50)	4 (57)
**Sex**						
Male	1,326 (62)	25 (71)	14 (64)	12 (80)	5 (63)	6 (86)
Female	824 (38)	10 (29)	8 (36)	3 (20)	3 (38)	1 (14)
**Period of illness onset**						
January–March	2 (<1)	0 (0)	1 (5)	0 (0)	0 (0)	0 (0)
April–June	88 (4)	4 (11)	6 (27)	1 (7)	1 (13)	0 (0)
July–September	1,781 (83)	25 (71)	3 (14)	11 (73)	7 (88)	6 (86)
October–December	279 (13)	6 (17)	12 (55)	3 (20)	0 (0)	1 (14)
**Clinical syndrome**						
Non-neuroinvasive	840 (39)	4 (11)	1 (5)	8 (53)	1 (13)	0 (0)
Neuroinvasive	1,310 (61)	31 (89)	21 (95)	7 (47)	7 (88)	7 (100)
Encephalitis	689 (32)	24 (69)	15 (68)	4 (27)	6 (75)	6 (86)
Meningitis	468 (22)	6 (17)	3 (14)	2 (13)	0 (0)	0 (0)
AFP^†^	78 (4)	0 (0)	1 (5)	0 (0)	0 (0)	0 (0)
Other	75 (3)	1 (3)	2 (9)	1 (7)	1 (13)	1 (14)
**Outcome**						
Hospitalization	1,465 (68)	32 (91)	20 (91)	7 (47)	8 (100)	7 (100)
Death	106 (5)	0 (0)	3 (14)	0 (0)	2 (25)	3 (43)

**Abbreviation:** AFP = acute flaccid paralysis.

* Three unspecified California serogroup virus cases were also reported.

^†^ Of the 78 West Nile virus disease patients with AFP, 44 (56%) also had encephalitis or meningitis; the additional AFP patient with Powassan virus also had encephalitis.

Among the 1,310 WNV neuroinvasive disease cases, 689 (53%) were reported as encephalitis, 468 (36%) as meningitis, 78 (6%) as acute flaccid paralysis, and 75 (6%) as other neurologic presentation. Of the 78 patients with reported acute flaccid paralysis, 44 (56%) also had reported encephalitis or meningitis. Among patients with neuroinvasive disease, 1,250 (95%) were hospitalized and 105 (8%) died. The incidence of neuroinvasive WNV disease in the United States was 0.41 per 100,000 population (Table 2). South Dakota (4.04 per 100,000), North Dakota (3.17), Nebraska (1.84), Wyoming (1.37), and Colorado (1.06) had the highest incidence rates (Table 2) (Figure).The largest number of cases were reported from California (335), Texas (252) and Illinois (98), which together accounted for just over half of neuroinvasive disease cases (52%). The incidence of WNV neuroinvasive disease increased with age, from 0.02 per 100,000 in children aged <10 years to 1.16 in adults aged ≥70 years. Incidence was higher among males (0.54 per 100,000) than among females (0.28).

**TABLE 2:** Number and rate* of reported cases of arboviral neuroinvasive disease, by virus type, U.S. Census division, and state — United States, 2016

U.S. Census division/State	Virus type											
	West Nile		La Crosse		Powassan		Jamestown Canyon		Saint Louis encephalitis		Eastern equine encephalitis	
	No.	Rate	No.	Rate	No.	Rate	No.	Rate	No.	Rate	No.	Rate
**United States**	**1,310**	**0.41**	**31**	**0.01**	**21**	**0.01**	**7**	**<0.01**	**7**	**<0.01**	**7**	**<0.01**
**New England**	15	0.10	—^†^	—	9	0.06	1	0.01	—	—	—	—
Connecticut	1	0.03	—	—	1	0.03	—	—	—	—	—	—
Maine	—	—	—	—	1	0.08	—	—	—	—	—	—
Massachusetts	10	0.15	—	—	5	0.07	1	0.01	—	—	—	—
New Hampshire	—	—	—	—	1	0.07	—	—	—	—	—	—
Rhode Island	2	0.19	—	—	1	0.09	—	—	—	—	—	—
Vermont	2	0.32	—	—	—	—	—	—	—	—	—	—
**Middle Atlantic**	43	0.10	—	—	1	<0.01	—	—	—	—	1	<0.01
New Jersey	11	0.12	—	—	—	—	—	—	—	—	1	0.01
New York	20	0.10	—	—	1	0.01	—	—	—	—	—	—
Pennsylvania	12	0.09	—	—	—	—	—	—	—	—	—	—
**East North Central**	177	0.38	12	0.03	5	0.01	5	0.01	1	<0.01	2	<0.01
Illinois	98	0.77	—	—	—	—	—	—	1	0.01	—	—
Indiana	15	0.23	—	—	—	—	—	—	—	—	—	—
Michigan	42	0.42	—	—	—	—	—	—	—	—	2	0.02
Ohio	12	0.10	9	0.08	—	—	—	—	—	—	—	—
Wisconsin	10	0.17	3	0.05	5	0.09	5	0.09	—	—	—	—
**West North Central**	175	0.81	3	0.01	5	0.02	1	<0.01	—	—	—	—
Iowa	16	0.51	—	—	—	—	—	—	—	—	—	—
Kansas	18	0.62	—	—	—	—	—	—	—	—	—	—
Minnesota	38	0.69	3	0.05	5	0.09	1	0.02	—	—	—	—
Missouri	9	0.15	—	—	—	—	—	—	—	—	—	—
Nebraska	35	1.84	—	—	—	—	—	—	—	—	—	—
North Dakota	24	3.17	—	—	—	—	—	—	—	—	—	—
South Dakota	35	4.04	—	—	—	—	—	—	—	—	—	—
**South Atlantic**	32	0.05	13	0.02	1	<0.01	—	—	—	—	3	<0.01
Delaware	—	—	—	—	—	—	—	—	—	—	—	—
District of Columbia	1	0.15	—	—	—	—	—	—	—	—	—	—
Florida	6	0.03	—	—	—	—	—	—	—	—	—	—
Georgia	5	0.05	—	—	—	—	—	—	—	—	1	0.01
Maryland	6	0.10	—	—	—	—	—	—	—	—	—	—
North Carolina	2	0.02	8	0.08	1^§^	0.01	—	—	—	—	2	0.02
South Carolina	6	0.12	—	—	—	—	—	—	—	—	—	—
Virginia	6	0.07	—	—	—	—	—	—	—	—	—	—
West Virginia	—	—	5	0.27	—	—	—	—	—	—	—	—
**East South Central**	48	0.25	3	0.02	—	—	—	—	—	—	—	—
Alabama	13	0.27	—	—	—	—	—	—	—	—	—	—
Kentucky	5	0.11	—	—	—	—	—	—	—	—	—	—
Mississippi	27	0.90	—	—	—	—	—	—	—	—	—	—
Tennessee	3	0.05	3	0.05	—	—	—	—	—	—	—	—
**West South Central**	319	0.80	—	—	—	—	—	—	—	—	—	—
Arkansas	8	0.27	—	—	—	—	—	—	—	—	—	—
Louisiana	38	0.81	—	—	—	—	—	—	—	—	—	—
Oklahoma	21	0.54	—	—	—	—	—	—	—	—	—	—
Texas	252	0.90	—	—	—	—	—	—	—	—	—	—
**Mountain**	156	0.65	—	—	—	—	—	—	3	0.01	1	<0.01
Arizona	57	0.82	—	—	—	—	—	—	—	—	—	—
Colorado	59	1.06	—	—	—	—	—	—	—	—	—	—
Idaho	3	0.18	—	—	—	—	—	—	—	—	—	—
Montana	3	0.29	—	—	—	—	—	—	—	—	1^§^	0.10
Nevada	13	0.44	—	—	—	—	—	—	2	0.07	—	—
New Mexico	6	0.29	—	—	—	—	—	—	—	—	—	—
Utah	7	0.23	—	—	—	—	—	—	1	0.03	—	—
Wyoming	8	1.37	—	—	—	—	—	—	—	—	—	—
**Pacific**	345	0.65	—	—	—	—	—	—	3	0.01	—	—
Alaska	—	—	—	—	—	—	—	—	—	—	—	—
California	335	0.85	—	—	—	—	—	—	3	0.01	—	—
Hawaii	—	—	—	—	—	—	—	—	—	—	—	—
Oregon	2	0.05	—	—	—	—	—	—	—	—	—	—
Washington	8	0.11	—	—	—	—	—	—	—	—	—	—

* Per 100,000 population, based on July 1, 2016, U.S. Census population estimates.

^†^ Dashes indicate none reported.

^§^ Patient reported travel to a state with a history of the virus.

**FIGURE:**
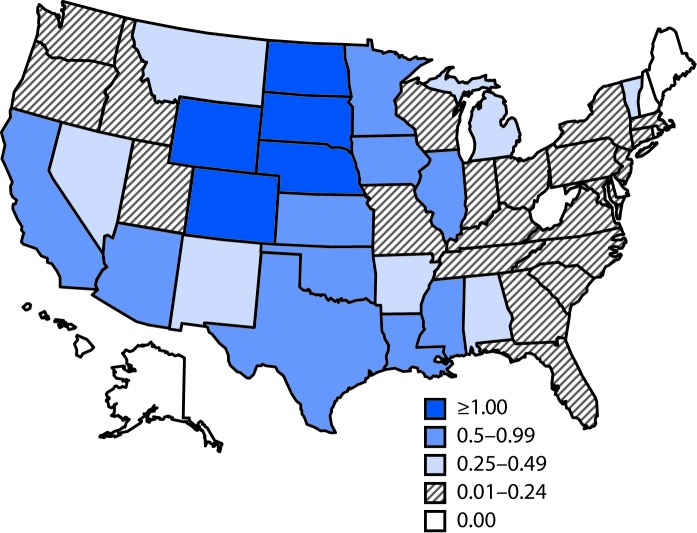
Rate* of reported cases of West Nile virus neuroinvasive disease — United States, 2016 * Per 100,000 population.

Thirty-five La Crosse virus disease cases were reported from six states (Minnesota, North Carolina, Ohio, Tennessee, Wisconsin, and West Virginia), including 31 (89%) that were neuroinvasive (Table 1). Illness onset ranged from May through October, with 25 (71%) patients reporting onset during July–September. Twenty-five (71%) patients were male. The median age was 9 years (IQR = 5–12 years) and 28 (80%) were aged <18 years. Thirty-two (91%) patients were hospitalized; none died. Among patients hospitalized, 29 (91%) had neuroinvasive disease. Incidence of La Crosse virus neuroinvasive disease was highest in West Virginia (0.27 per 100,000) (Table 2).

Twenty-two Powassan virus disease cases were reported from nine states (Connecticut, Maine, Massachusetts, Minnesota, New Hampshire, New York, North Carolina, Rhode Island, and Wisconsin). Illness onset dates ranged from February through December. The median age of patients was 66 years (IQR = 61–72 years) and 14 (64%) were male. Twenty-one (95%) cases were neuroinvasive. Twenty (91%) patients were hospitalized and three (14%) died.

Fifteen Jamestown Canyon virus disease cases were reported from three states (Massachusetts, Minnesota, and Wisconsin). Illness onset dates ranged from June through November, with 11 (73%) of those patients reporting onset during July–September. The median age of patients was 64 years (IQR = 44–70 years) and 12 (80%) were male. Seven (47%) cases were neuroinvasive, seven (47%) patients were hospitalized, and none died.

Eight cases of St. Louis encephalitis virus disease were reported from four states (California, Illinois, Nevada, and Utah). The median age of patients was 64 years (IQR = 56–74) and five (63%) were male. Illness onset dates ranged from June through September. Seven (88%) cases were neuroinvasive (Table 1). All eight patients were hospitalized and two (25%) died.

Seven cases of eastern equine encephalitis virus disease were reported from five states (Georgia, Michigan, Montana, New Jersey, and North Carolina); all were neuroinvasive disease. The median age of patients was 63 years (IQR = 39–71 years) and six (86%) were male. Illness onset dates ranged from July through October. All patients were hospitalized and three (43%) died.

## Discussion

As in previous years, in 2016, WNV remained the most common cause of neuroinvasive arboviral disease in the continental United States, accounting for 95% of reported neuroinvasive disease cases. The incidence of WNV neuroinvasive disease in 2016 (0.41 per 100,000) was the same as the median incidence during 2002–2015 (*2*). The case fatality rate for neuroinvasive disease cases (8%) was comparable to that reported in past years (median of 9% for 1999–2015).

La Crosse virus continued to be more frequently reported in children than in other age groups (*5*). Overall, however, fewer cases of La Crosse virus were reported in 2016 than in any year in the past decade. More cases of Powassan virus were reported in 2016 than in previous years (22 in 2016 compared with a median of seven cases each year during 2006–2015) (*6*). This increase was, in part, likely caused by increased awareness and testing for the virus. In 2016, Powassan virus disease was reported for the first time in Connecticut and Rhode Island (*6*). The patient from North Carolina had history of travel to a state with previously documented Powassan virus transmission. Three states (California, Illinois, and Utah) reported cases of St. Louis encephalitis virus disease for the first time in >10 years. However, fewer cases were reported than in 2015, a year in which an outbreak in Arizona occurred (*7*). Eastern equine encephalitis virus was again the domestic arboviral disease with the highest fatality rate, with four deaths reported among the seven patients with neuroinvasive disease. Cases were reported from states that have historically reported eastern equine encephalitis virus, with the exception of a case from Montana, where the infection was acquired in a state with previously documented transmission.

Arboviruses continue to cause substantial morbidity in the United States, although reported numbers of cases vary annually. Cases occur sporadically, and the epidemiology varies by virus and geographic area. Consistent with past years, in 2016 just over 85% of arboviral disease cases occurred during April–September. Weather, zoonotic host and vector abundance, and human behavior are all factors that can influence when and where outbreaks occur. These factors make it difficult to predict future locations and timing of cases and help to emphasize the importance of surveillance to identify outbreaks and inform public health prevention efforts.

The findings in this report are subject to at least two limitations. First, ArboNET is a passive surveillance system, which leads to an underestimation of the actual prevalence of disease. To be reported as a disease case, the person affected must seek care, a clinician must request appropriate diagnostic tests, and health care providers and laboratories must then report cases to public health authorities. Previous studies have estimated that between 30 and 70 non-neuroinvasive disease cases occur for every reported case of WNV neuroinvasive disease (*8*–*10*). Based on the number of neuroinvasive disease cases reported in 2016, between 39,300 and 91,700 non-neuroinvasive disease cases of WNV would have been expected to occur; however, only 840 (1%–2%) were reported. Second, because ArboNET does not require information about clinical signs and symptoms or laboratory findings, cases might be misclassified.

It is important for health care providers to consider arboviral infections in the differential diagnosis of cases of aseptic meningitis and encephalitis, obtain appropriate specimens for laboratory testing, and promptly report cases to public health authorities (*2*). Understanding the epidemiology, seasonality, and geographic distribution of these viruses will assist with clinical recognition and potential differentiation from other neuroinvasive etiologies. Because human vaccines against domestic arboviruses are not available, prevention depends on community and household efforts to reduce vector populations (e.g., applying insecticides and reducing breeding sites), personal protective measures to decrease exposure to mosquitoes and ticks (e.g., use of repellents and wearing protective clothing), and screening of blood donors.

**Summary**

**What is already known about this topic?**

Arboviral disease can cause considerable morbidity and mortality in the United States. West Nile virus (WNV) is consistently found to be the leading cause of domestically acquired arboviral disease, but several other arboviruses cause sporadic cases and outbreaks of neuroinvasive disease.

**What is added by this report?**

In 2016, WNV continued to be the most common cause of neuroinvasive arboviral disease in the United States, with a similar incidence to the median incidence during 2002–2015. An increase in reported cases of Powassan virus occurred in 2016, with two states reporting their first cases.

**What are the implications for public health practice?**

Arboviral diseases are a continuing source of severe illness each year. Surveillance remains important to identify outbreaks and guide prevention strategies.
